# Current and Potential Pharmacologic Therapies for Traumatic Brain Injury

**DOI:** 10.3390/ph15070838

**Published:** 2022-07-06

**Authors:** Jowy Tani, Ya-Ting Wen, Chaur-Jong Hu, Jia-Ying Sung

**Affiliations:** 1 Department of Neurology, Wan Fang Hospital, Taipei Medical University, Taipei 116, Taiwan; jowytani@tmu.edu.tw; 2 Sleep Medicine Center, Wan Fang Hospital, Taipei Medical University, Taipei 116, Taiwan; 3 Biomed Innovation Center, Wan Fang Hospital, Taipei Medical University, Taipei 116, Taiwan; 4 Department of Neurology, School of Medicine, College of Medicine, Taipei Medical University, Taipei 110, Taiwan; chaurjongh@tmu.edu.tw; 5 Taipei Medical University Biomed Accelerator, Taipei Medical University, Taipei 106, Taiwan; 6 Taipei Medical University Biodesign Center, Taipei Medical University, Taipei 106, Taiwan; 7 Taipei Neuroscience Institute, Taipei Medical University, Taipei 110, Taiwan; 98142@w.tmu.edu.tw; 8 Department of Neurosurgery, Wan Fang Hospital, Taipei Medical University, Taipei 116, Taiwan; 9 Department of Neurosurgery, School of Medicine, College of Medicine, Taipei Medical University, Taipei 110, Taiwan; 10 Department of Neurology, Shuang Ho Hospital, Taipei Medical University, New Taipei City 235, Taiwan

**Keywords:** traumatic brain injury, pharmacologic therapies, psychopharmacology, neuroprotectants

## Abstract

The present article reviewed the pharmacologic therapies of traumatic brain injury (TBI), including current and potential treatments. Pharmacologic therapies are an essential part of TBI care, and several agents have well-established effects in TBI care. In the acute phase, tranexamic acid, antiepileptics, hyperosmolar agents, and anesthetics are the mainstay of pharmacotherapy, which have proven efficacies. In the post-acute phase, SSRIs, SNRIs, antipsychotics, zolpidem and amantadine, as well as other drugs, have been used to manage neuropsychological problems, while muscle relaxants and botulinum toxin have been used to manage spasticity. In addition, increasing numbers of pre-clinical and clinical studies of pharmaceutical agents, including potential neuroprotective nutrients and natural therapies, are being carried out. In the present article, we classify the treatments into established and potential agents based on the level of clinical evidence and standard of practice. It is expected that many of the potential medicines under investigation will eventually be accepted as standard practice in the care of TBI patients.

## 1. Introduction

**Figure 1:**
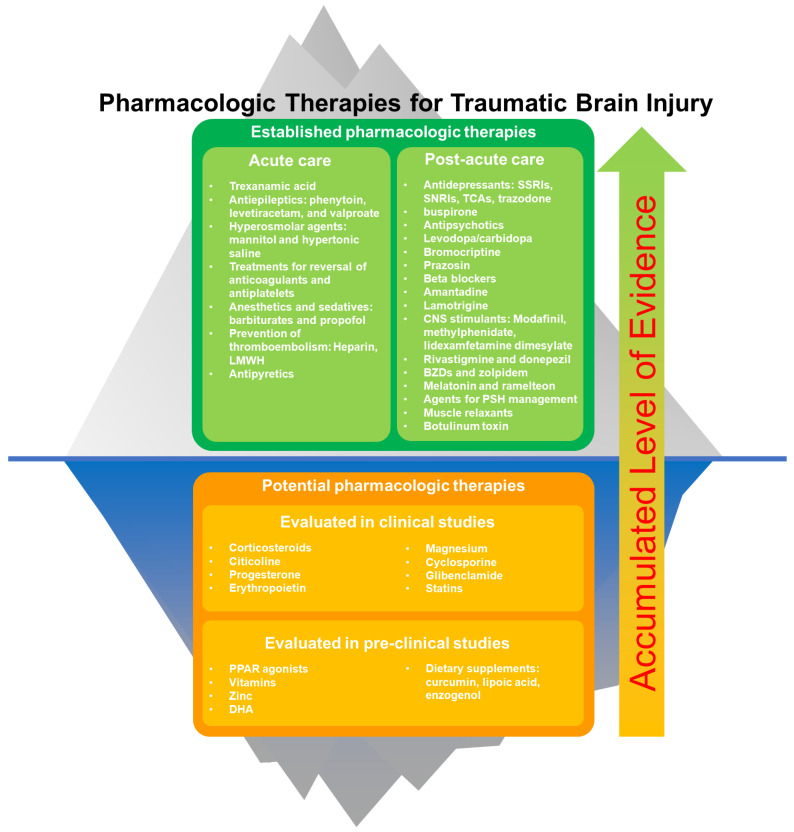
Established and potential pharmacologic therapies for traumatic brain injury. Potential pharmaceutical agents may be accepted into standard practice (“go above the surface”) with the accumulation of clinical evidence.

Traumatic brain injury (TBI) is a sudden injury that causes damage to the brain. Sixty-nine million individuals worldwide are estimated to sustain a TBI each year [1]. Pharmacologic therapies play important roles in mild to severe TBI. There are several pharmacologic therapies recommended by guidelines, which have proven efficacies and well-documented safety profiles for use in acute and post-acute TBI patients [2]. In addition, several new preclinical and clinical studies of pharmacologic therapies for TBI have been published recently, which could contribute to the addition of new agents into standard TBI management in the future. This review discusses current and potential pharmacologic therapies for TBI. We also summarize the pharmacologic therapies for TBI in Figure 1.

## 2. Established Pharmacologic Therapies for TBI

Following traumatic brain injury, primary damage results from mechanical damage affecting cells and tissue. Hemorrhage and breakdown of the blood–brain barrier (BBB) also happen within seconds to minutes. Secondary damage develops within minutes, with the development of inflammation, ischemia and edema [3].

Subsequent processes, including delayed inflammation, vasospasm, cell death and genomic responses, develop within days. Cellular degeneration, neuropsychiatric comorbidities and muscle spasticity are noted over the next few weeks to months. Recent advances in biomarkers, including microRNA, have enhanced our understanding of the pathophysiologic process and could even help researchers to determine the time elapsed since an injury [3,4,5]. In current practice, pharmacological therapies are provided to treat both the acute and chronic effects of these pathological processes.

Published literature studying the effects of biological sex and gender shows mixed outcomes on TBI. A recent review of the literature found that women, after puberty but before menopause, were at higher risk of poor outcome, while postmenopausal women fared better than men of similar age [6]. Care pathways and treatment probably did not differ significantly between women and men [7]. More study is needed to support treatment strategies for different sexes.

### 2.1. Acute Treatments for TBI

#### 2.1.1. Tranexamic Acid

Tranexamic acid reduces the risk of death in mild to moderate TBI patients when treatment is given within 3 h, in a loading dose of 1 g, followed by infusion of 1 g for 8 h, according to a recent CRASH-3 trial [8]. However, tranexamic acid does not reduce death in severe TBI patients who have extensive intracranial hemorrhage.

#### 2.1.2. Treatments for Coagulopathies

Around one-third of severe TBI patients demonstrate coagulopathy, which may lead to hemorrhage enlargement and poor neurologic outcomes. Coagulopathy mostly results from existing medications, such as aspirin, clopidogrel, direct oral anticoagulants or warfarin. It has been demonstrated that direct oral anticoagulants do not increase the incidence of intracranial hemorrhage [9], and there are better outcomes for direct oral anticoagulant use compared to warfarin use, even with low use of the reversal strategy [10,11].

Patients taking warfarin could be managed with vitamin K and fresh frozen plasma (FFP) infusion, monitoring prothrombin time/international normalized ratio (INR) 30 min after transfusion or every 4 to 6 h to ensure INR < 1.4 [12].

In patients using anti-platelet agents or thrombocytopenia, a platelet count > 95,000/μL directly with platelet transfusion should be maintained. In one cohort study, a platelet count < 135,000/μL was associated with a 12.4 times higher risk of hemorrhage enlargement; patients with a platelet count < 95,000/μL were 31.5 times more likely to require neurosurgical intervention [13].

#### 2.1.3. Hyperosmolar Agents

Mannitol and hypertonic saline are commonly used in the management of intracranial hypertension and cerebral edema. Mannitol at bolus doses of 0.25–1 g/kg every 4 to 6 h is effective in reducing brain volume, and thus lowering intracranial pressure (ICP) [2]. However, its diuretic effect should be monitored cautiously in hypotensive patients. Mannitol is not recommended in patients with systolic blood pressure < 90 mm Hg.

Hypertonic saline is also an effective hyperosmolar agent for lowering increased ICP [14,15]. Infusion of 3% hypertonic saline is administrated to achieve a sodium level goal of 145–155 mEq/L. There is less volume depletion and hypovolemia, which makes hypertonic saline safer in major trauma patients with ongoing volume loss and hypotension. When comparing these two hyperosmolar agents, there was no strong evidence to suggest the superiority of either in improving mortality or functional recovery [16,17].

#### 2.1.4. Anesthetics and Sedatives

Anesthetics and sedatives are commonly used in acute stage TBI management in the intensive care unit (ICU) setting. Barbiturates and propofol have both been shown to depress cerebral metabolism, decrease oxygen consumption, lower ICP and prevent seizures. They are recommended as adjuvant therapy to control elevated ICP when refractory to maximum hyperosmolar therapy and surgical decompression. However, hemodynamic stability should be monitored during barbiturate or propofol therapy. Barbiturates result in a decrease in blood pressure in 25% of patients [18]. Body temperature is also significantly lower. Therefore, the duration and dose of barbiturate administration need to be carefully observed. It could be used under continuous monitoring of EEG to achieve optimal doses.

Within the ICU, propofol is even more widely used in acute TBI management. It is easier to control the treatment effects because of its rapid onset and short duration characteristics. However, caution is required, as high-dose propofol can result in morbidity. Propofol infusion syndrome could lead to hyperkalemia, metabolic acidosis, hyperlipidemia, myocardial failure and renal failure, which may result in death. Therefore, extreme caution must be taken when using propofol doses > 4 mg/kg/h, or when use exceeds 48 h. For refractory ICP elevation, pentobarbital and thiopental infusions may be used [18]. Nevertheless, the therapy may delay timely neurologic examination. It may also result in hypotension, ileus, ventilator-associated pneumonia and metabolic acidosis.

#### 2.1.5. Drugs for Prevention of Thromboembolism

Heparin or low-molecular-weight heparin (LMWH) for the prevention of venous thromboembolism in TBI patients is generally safe if initiated within 24–48 h of injury [19].

#### 2.1.6. Antiepileptics

The general incidence of post-traumatic seizure in hospitalized populations of TBI is about 3–5% [20,21]. In a study enrolling 5984 TBI patients in Minnesota from 1935 to 1984, the incidence of seizures ranged between 0.7 and 10% in five years of follow-up, correlating with the severity of TBI [22]. The use of antiepileptic drugs in the acute management of TBI has been proven to reduce the incidence of early seizures, but does not prevent the later development of epilepsy.

Furthermore, subclinical seizures detected from an electroencephalogram may be as high as 20–25% [23]. Thus, it is recommended to use prophylactic antiepileptic drugs to avoid early seizures after TBI (within 7 days of injury) [24]. Antiepileptics are recommended in the first seven days following injury in guidelines. Continued use of antiepileptics is recommended if there are electroencephalogram (EEG) discharges. Use of antiepileptics prevents post-traumatic seizures, but does not prevent later development of epilepsy [25,26].

Carbamazepine and valproate are also used as mood stabilizers for psychomotor aggregation after TBI, but the effects are controversial [27]. A newer antiepileptic drug, levetiracetam, is commonly used as there is less drug interaction and it has an equal effect as phenytoin in preventing early seizures [28]. The optimal duration of prophylactic antiepileptic drugs is uncertain and depends on the severity of brain injury. In the absence of early seizures, antiepileptic drugs are usually continued throughout the hospital stay and are discontinued within the first few weeks of discharge [29,30].

A recent meta-analysis study at World Neurosurgery, comparing the efficacy of phenytoin, levetiracetam and valproate in preventing early seizures in TBI patients, showed that phenytoin was the most studied drug. Phenytoin has level 2a evidence to decrease the incidence of early post-traumatic seizures [31]. However, more studies are needed to assess the efficacy of other antiepileptic drugs, such as levetiracetam and valproate. Currently, there is insufficient evidence to recommend levetiracetam or valproate over phenytoin. 

#### 2.1.7. Antipyretics

Fever could lead to worse outcomes after TBI, and antipyretics could be used to control fever in acute TBI. Maintenance of normothermia also improves ICP control [32] and brain tissue oxygenation [33].

### 2.2. Treatments for Post-TBI Neuropsychiatric Changes

Neuropsychiatric changes following TBI could cause significant distress in patients and long-term disability [34]. The choice of pharmacologic treatments could have a significant impact on post-acute TBI care, as well as the patient’s neurological recovery [35]. The present review further discusses pharmaceutical agents that have been studied for their use in post-acute TBI.

#### 2.2.1. SSRIs and SNRIs

Several studies have been conducted on the efficacies of various selective serotonin reuptake inhibitors (SSRIs) in the treatment of depression. Sertraline [36], citalopram [36,37], and fluoxetine [36] have been shown to be beneficial in the treatment of post-TBI depression. Sertraline could even potentially prevent the later onset of depression [38,39]. Sertraline probably does not improve arousal in TBI patients [40].

SSRIs [41], including citalopram [42], sertraline [43] and paroxetine [44], could improve post-TBI pathological laughing and crying. Fluvoxamine and fluoxetine could probably improve apathy [45].

Serotonin and norepinephrine re-uptake inhibitors (SNRIs), such as milnacipran, have also been shown to be efficacious in the treatment of depression [46]. Another SNRI, atomoxetine, has failed to improve attention, speed of memory or working memory, compared to a placebo [47].

#### 2.2.2. Trazodone

Trazodone may cause impaired attention and errors with memory tests [48], but has mixed results on sleep [49].

#### 2.2.3. TCAs

Desipramine has been shown to improve depression in severe TBI [50]. However, tricyclic antidepressants (TCAs) are probably less effective than SSRIs in the treatment of post-TBI depression [51,52], and are associated with more complications [53].

#### 2.2.4. Buspirone

Buspirone is a serotonin 1A receptor partial agonist that has been shown to reduce anxiety in patients with TBI [54].

#### 2.2.5. Antipsychotics

Typical antipsychotics, including methotrimeprazine [55], droperidol, haloperidol [56] and loxapine [57], could improve agitation. Atypical antipsychotics, including quetiapine [58], clozapine [59], ziprasidone [60] and aripiprazole [61], have also been shown to improve agitation. Olanzapine has been shown to improve post-TBI psychosis [62,63]. Atypical antipsychotics are generally preferred over typical antipsychotics in post-TBI patients, due to their more favorable profile in safety and neurorecovery [64].

#### 2.2.6. Levodopa/Carbidopa

Levodopa/carbidopa has been shown to improve consciousness [65].

#### 2.2.7. Bromocriptine

Bromocriptine is a direct dopamine agonist at the D2 receptor. It could improve arousal [66], but probably could not improve attention [67].

#### 2.2.8. Prazosin

Prazosin has been shown to reduce daytime sleepiness, improve headaches and improve cognition [68].

#### 2.2.9. Beta Blockers

Beta blockers, such as propranolol and pindolol, have been shown to reduce post-TBI agitation in some studies [69]. Nevertheless, their hypotensive effect may limit the dose that could be applied.

#### 2.2.10. Amantadine

Amantadine has been shown to improve the pace of functional recovery, as measured by the Disability Rating Scale (DRS) [70]. It has also been shown to improve early arousal in the acute phase of TBI [71,72,73].

#### 2.2.11. Lamotrigine

Lamotrigine has been shown to reduce aggressive behavior in TBI patients [74].

#### 2.2.12. Modafinil and Methylphenidate

Modafinil could probably improve excessive daytime sleepiness [75], but probably does not improve fatigue [76]. It might be able to improve sleep latency in patients with mild or moderate TBI [77]. Methylphenidate has been shown to improve post-TBI attention and processing speed [78,79,80,81].

#### 2.2.13. Lisdexamfetamine Dimesylate

Lisdexamfetamine dimesylate has been shown to improve attention and working memory in a small-scale study [82].

#### 2.2.14. Rivastigmine and Donepezil

Rivastigmine and donepezil are well known for their use in the treatment of Alzheimer’s disease. Donepezil is currently undergoing clinical studies to confirm its effect on memory, attention and processing speed [83]. Rivastigmine did not appear to improve cognition significantly [84], but it showed some improvement in memory in some subgroups of patients in a post hoc analysis of one study [85]. 

#### 2.2.15. Benzodiazepines and Zolpidem

Benzodiazepines are associated with attentional and memory impairments in TBI, and are generally to be avoided [86,87]. They may impair coordination, leading to falls, increase sedation, negatively affect memory [49], and they may also lead to sleep–wake disturbances [88].

Interestingly, zolpidem has been shown to cause a temporary response in a fraction of patients with severe TBI [89]. It could probably cause attenuation of inter-hemispheric coherences on electroencephalograms [90], and improved cerebral perfusion was observed on single-photon emission computed tomography (SPECT) [91].

#### 2.2.16. Melatonin and Ramelteon

Melatonin might be able to improve daytime sleepiness in TBI patients [92]. Ramelteon has been shown to improve total sleep time and could potentially improve cognition [93].

### 2.3. Other Pharmaceutical Agents for Post-Acute TBI Care

#### 2.3.1. Muscle Relaxants

Spasticity is an important problem, particularly in moderate and severe TBI. Oral baclofen could improve the lower extremity Modified Ashworth Score [94]. Intrathecal baclofen might be able to improve muscle spasms even more than oral baclofen [95]. Oral tizanidine has been shown to reduce the Ashworth score, enhance motor strength and reduce muscle tone [96].

#### 2.3.2. Botulinum Toxin

A botulinum toxin injection might also be beneficial in the treatment of spasticity in TBI patients [97]. Botulinum toxin might also improve chronic post-traumatic headache [98].

#### 2.3.3. Agents for Paroxysmal Sympathetic Hyperactivity Management

The various drugs discussed above are used for the prevention and/or abortion of paroxysmal sympathetic hyperactivity (PSH), which occurs in up to 10% of patients with severe TBI. Drugs that have been studied for the treatment of PSH in TBI include beta blockers, benzodiazepines, bromocriptine [99], dantrolene [100] and gabapentin [101].

## 3. Potential Therapies for TBI

### 3.1. Neuroprotective Approaches Previously Evaluated in Clinical Studies

Several pharmaceutical agents have been evaluated in clinical studies for their potential efficacies in the treatment of TBI. So far, the routine use of most of these agents in the management of TBI has not been justified. Nevertheless, future evidence may arise to support their use in the care of TBI patients.

#### 3.1.1. Corticosteroids

Corticosteroid was one of the first agents studied for its neuroprotective effect in TBI. The use of corticosteroids has been studied in the Medical Research Council’s Corticosteroid Randomization after Significant Head Injury study [102,103]. This large-scale study found that treatment with glucocorticoids increased mortality.

#### 3.1.2. Citicoline

Citicoline is a cholinergic agent that increases the formation of ATP. It was evaluated in a multi-center, double-blind, randomized phase III controlled trial, The Citicoline Brain Injury Treatment Trial (COBRIT), but it did not improve outcomes [104].

#### 3.1.3. Progesterone

Despite the potential benefits shown in two older, small-scale studies [105,106], progesterone has been evaluated by two large-scale clinical trials: SyNAPSe and ProTECT III [107,108], but did not demonstrate clinical benefit in patient mortality and functional outcomes. Some clinical studies suggested that progesterone might be neuroprotective [109,110].

#### 3.1.4. Erythropoietin

One randomized controlled trial showed that erythropoietin treatment results in lower mortality, but that result is not statistically significant [111]. Two meta-analyses of trials also suggested that erythropoietin might lower mortality, but not reduce poor functional outcomes [112,113]. Other studies have not revealed evidence of improved outcomes from erythropoietin use [114].

#### 3.1.5. Magnesium

The use of magnesium has been evaluated in a number of heterogeneous clinical studies [115,116]. A meta-analysis concluded that while all-cause mortality did not improve in the treatment group, the GCS might have improved [115].

#### 3.1.6. Cyclosporine

Cyclosporine has been evaluated in a few small-scale clinical trials, and did not appear to contribute to a favorable outcome [117,118].

#### 3.1.7. Glibenclamide

Glibenclamide is an antagonist of sulfonylurea receptor 1 (SUR1). It has been evaluated in several small-scale clinical studies and showed favorable outcomes, such as an improved Glasgow Coma Scale (GCS) score and improved Glasgow Outcome Scale (GOS) score [119,120,121,122].

#### 3.1.8. Statins

Clinical studies of statins in TBI patients suggested that statin use might improve functional outcomes. It might also lead to a reduction in pro-inflammatory mediators [123,124,125].

### 3.2. Neuroprotective Approaches and Natural Therapies Previously Evaluated in Pre-Clinical Studies

#### 3.2.1. PPAR Agonists

Peroxisome proliferator-activated receptor (PPAR) agonists, such as rosiglitazone pioglitazone, play a role in the regulation of gene transcriptions, which are essential in metabolic processes and cell differentiation. Its neuroprotective properties were suggested in several pre-clinical studies [126]. It might exert such an effect by decreasing axonal injury, decreasing apoptosis, decreasing autophagy and/or decreasing microglial activation [126,127,128].

#### 3.2.2. Vitamins

Vitamin D could reduce inflammation biomarkers and prevent neuron death in animal models when it is used together with progesterone [129]. Vitamin E has been reported to enhance the neuroprotective effects of progesterone [130]. Water-soluble nicotinamides aggregate the functional recovery of TBI rodents [131,132]. An equivocal effect of folic acid has been documented [133,134]. However, there is an overall lack of clinical trials on vitamins for TBI patients.

#### 3.2.3. Zinc

Zinc has been shown to have double effects on both anti-inflammation and anti-oxidative damage [135]. High zinc supplements might decrease neuropsychiatric symptoms in TBI patients, based on animal experiment results [136,137,138].

#### 3.2.4. DHA

Docosahexaenoic (DHA) is a fatty acid that exists in phospholipids of the neuron membrane. It can be released to counteract glutamate overactivity after brain damage [139]. DHA can also reduce endoplasmic reticulum (ER) stress and prevent abnormal protein accumulation in the TBI model [140]. DHA is quite a safe and accessible food supplement and might be beneficial for neuroprotection in traumatic brain injury; however, human clinical studies are necessary.

#### 3.2.5. Dietary Supplements

Curcumin has been reported to improve the motor and learning ability in TBI animal models [141]. Resveratrol has been shown to reduce reactive oxygen species (ROS), inhibit excitotoxicity and decrease inflammation in cortical injury models of TBI [142]. Lipoic acid could stabilize plasma membranes and prevent NADPH (nicotinamide adenine dinucleotide phosphate) oxidative stress in mild TBI rats. In a clinical trial [143], Enzogenol has been shown to take advantage of the cognitive function in TBI patients. Both nutrients and pharmacological treatment are important for the recovery of TBI. A low nutrient intake in TBI is correlated with poor outcomes [144].

## 4. Conclusions

**Figure 2:**
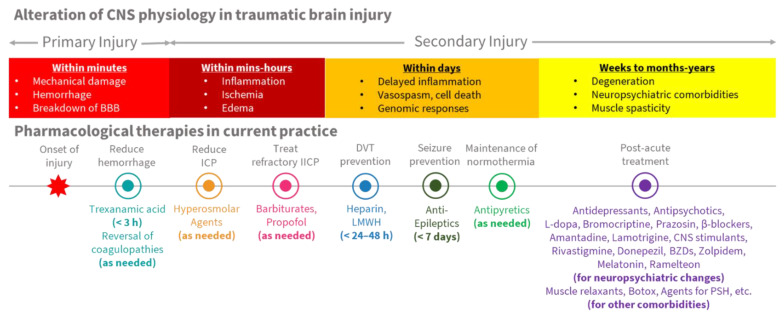
Alteration of CNS physiology and pharmacological therapies in TBI.

Figure 2 shows the use of each type of pharmacologic agent, in various phases of alteration within central nervous system (CNS) physiology, following traumatic brain injury. Table 1 shows current pharmaceutical therapies for TBI, based on the timing of use, and main effects on the CNS.

Various pharmacological treatments could affect the pathophysiology of TBI; proper treatment can reduce the detrimental effect of brain trauma in the acute and post-acute phases, and improve the overall prognosis. In this review, we have summarized medications based on clinical evidence and usage, though more clinical studies should be carried out for potential pharmacologic therapies. We expect that the accumulation of clinical evidence on newer agents would eventually lead to new therapeutic strategies that eventually improve the quality of TBI care.

## Figures and Tables

**Table 1:** Current pharmaceutical therapies for TBI.

Pharmaceutical Agents	Effects on CNS	Timing of Usage	Role in TBI Treatment	Reference
Acute phase				
Tranexamic acid	Antifibrinolytics	Within 3 h of injury	Reduces the risk of death in mild to moderate TBI	[8]
Vitamin K, FFP, Direct oral anticoagulants reversal agents	Coagulopathy reversal agents	Immediately after coagulopathy is identified	Reversal of coagulopathies	[9,10,11,12,13]
Mannitol and hypertonic saline	Elevates blood plasma osmolality, drawing water from brain and CSF	When impending cerebral herniation is noted; assessment in every 1–2 h	Management of intracranial hypertension and cerebral edema	[16,17]
Barbiturates and propofol	Anesthetics and sedatives	When there is elevated ICP refractory to other therapies	Depress cerebral metabolism, decreased oxygen consumption, lower ICP, and prevent seizures	[18]
Heparin, LMWH	Anticoagulants	Within 24–48 h of injury	Prevention of venous thromboembolism	[19]
Phenytoin, levetiracetam, and valproate	Antiepileptics	Within 7 days of injury	Reduce the incidence of early seizures but does not prevent the later development of epilepsy	[31]
Paracetamol and NSAIDs	Antipyretics	If fever	Maintenance of normothermia	[32,33]
**Post-acute phase**				
SSRIs	Block the reabsorption of serotonin into neurons.	Weeks to months	Improve post-TBI depression, apathy, pathological laughing and crying; prevent the later onset of depression	[36,37,38,39,41,42,43,44,45,145]
SNRIs	Block the reuptake of serotonin and noradrenaline	Weeks to months	Improve post-TBI depression	[46,47]
Trazodone	Serotonin antagonist and reuptake inhibitor	Weeks to months	Mixed results on sleep	[49]
TCAs	Block the reuptake of serotonin and norepinephrine	Weeks to months	Treatment of post-TBI depression	[50]
Buspirone	Agonist of 5-HT1A receptor	Weeks to months	Reduce anxiety in patients with TBI	[54]
Typical and atypical antipsychotics	Block the dopamine receptors	Weeks to months	Improve post-TBI psychosis	[55,56,57,58,59,60,61,62,63]
Levodopa/carbidopa	Agonist of Dopamine receptor	Weeks to months	To improve consciousness	[65]
Bromocriptine	Agonist of the D2 receptor	Weeks to months	To improve arousal	[66]
Prazosin	Block the α1 receptor	Weeks to months	Reduce daytime sleepiness, improve headaches, and improve cognition	[68]
Beta blockers	Block the β-adrenergic receptors	Weeks to months	Reduce post-TBI agitation	[69]
Amantadine	Antagonist of the NMDA-type glutamate receptor	Weeks to months	Improve the pace of functional recovery. Improve early arousal in the acute phase of TBI	[37,71,72,73]
Lamotrigine	Sodium channel blocker	Weeks to months	Reduce aggressive behavior in TBI patients	[74]
Modafinil	Central nervous system stimulant	Weeks to months	Could improve excessive daytime sleepiness and sleep latency	[75,77]
Methylphenidate	Central nervous system stimulant	Weeks to months	Could improve post-TBI attention and processing speed	[78,79,80,81]
Lisdexamfetamine dimesylate	Central nervous system stimulant	Weeks to months	improve attention and working memory	[82]
Rivastigmine and donepezil	Inactivate the cholinesterases	Weeks to months	Could improve memory in some subgroups of patients	[85]
Benzodiazepines	Agonist of GABA receptor	Weeks to months	Generally to be avoided; may impair attention, coordination, memory, and increase sedation	[86,87]
Zolpidem	Agonist of GABA receptor	Weeks to months	Could cause a temporary response in a fraction of patients with severe TBI	[89]
Melatonin	Agonist of melatonin receptors	Weeks to months	Improve daytime sleepiness.	[92]
Ramelteon	Agonist of melatonin receptors	Weeks to months	Could improve total sleep time	[93]
Balofen, tizanidine	Block transmission at the neuromuscular junction	Weeks to months	Decrease muscle spasm, reduce muscle tone	[94,95,96]
Botulinum toxin	Block presynaptic release of the acetylcholine at the neuromuscular junction	Weeks to months	Treatment of spasticity, improve post-TBI headache	[97,98]

## Data Availability

Not applicable.
